# PGSE Is a Novel Enhancer Regulating the Proteoglycan Pathway of the Mammalian Golgi Stress Response

**DOI:** 10.1247/csf.18031

**Published:** 2018-11-28

**Authors:** Kanae Sasaki, Ryota Komori, Mai Taniguchi, Akie Shimaoka, Sachiko Midori, Mayu Yamamoto, Chiho Okuda, Ryuya Tanaka, Miyu Sakamoto, Sadao Wakabayashi, Hiderou Yoshida

**Affiliations:** 1 Department of Molecular Biochemistry, Graduate School of Life Science, University of Hyogo, Hyogo 678-1297, Japan

**Keywords:** Golgi stress, proteoglycan, ER stress, organelle zone, organelle autoregulation

## Abstract

The Golgi stress response is a homeostatic mechanism that augments the functional capacity of the Golgi apparatus when Golgi function becomes insufficient (Golgi stress). Three response pathways of the Golgi stress response have been identified in mammalian cells, the TFE3, HSP47 and CREB3 pathways, which augment the capacity of specific Golgi functions such as N-glycosylation, anti-apoptotic activity and pro-apoptotic activity, respectively. On the contrary, glycosylation of proteoglycans (PGs) is another important function of the Golgi, although the response pathway upregulating expression of glycosylation enzymes for PGs in response to Golgi stress remains unknown. Here, we found that expression of glycosylation enzymes for PGs was induced upon insufficiency of PG glycosylation capacity in the Golgi (PG-Golgi stress), and that transcriptional induction of genes encoding glycosylation enzymes for PGs was independent of the known Golgi stress response pathways and ER stress response. Promoter analyses of genes encoding these glycosylation enzymes revealed the novel enhancer elements PGSE-A and PGSE-B (the consensus sequences are CCGGGGCGGGGCG and TTTTACAATTGGTC, respectively), which regulate their transcriptional induction upon PG-Golgi stress. From these observations, the response pathway we discovered is a novel Golgi stress response pathway, which we have named the PG pathway.

## Introduction

Eukaryotic cells have sets of different organelles that share specific functions. The functional capacity of each organelle is tightly regulated in accordance with cellular demands by organelle autoregulation mechanisms, which is one of the critical issues in cell biology (Sasaki and Yoshida, 2015). The autoregulatory mechanisms regulating the capacity of the endoplasmic reticulum (ER) and the Golgi apparatus are the ER stress response (Amin-Wetzel *et al.*, 2017; Han and Kaufman, 2017; Ishikawa *et al.*, 2017; Karagoz *et al.*, 2017; Tsuchiya *et al.*, 2018) and the Golgi stress response (Baumann *et al.*, 2018; Miyata *et al.*, 2013; Taniguchi and Yoshida, 2017), respectively, and other organelles have their own autoregulatory mechanisms (see Discussion). The molecular mechanism of the ER stress response has been well characterized. The mammalian ER stress response consists of three distinct response pathways: the ATF6, IRE1 and PERK pathways. The ATF6 pathway regulates expression of ER chaperone genes (Okada *et al.*, 2002; Yamamoto *et al.*, 2007), whereas the IRE1 pathway augments expression of factors for ER-associated degradation (Calfon *et al.*, 2002; Cox *et al.*, 1993; Mori *et al.*, 1993; Shen *et al.*, 2001; Yoshida *et al.*, 2003, 2001). The PERK pathway controls ER stress-induced apoptosis (Harding *et al.*, 2000, 1999; Yan *et al.*, 2002). Each pathway has a sensor that detects ER stress, as well as a transcription factor that activates transcription of ER-related genes through a specific enhancer element. In the case of the ATF6 pathway, the sensor, the transcription factor and the enhancer element are pATF6(P), pATF6(N) and ERSE, respectively (Haze *et al.*, 1999; Yoshida *et al.*, 1998, 2000, 2001), and those of the IRE1 pathway are IRE1, pXBP1(S) and UPRE, respectively (Calfon *et al.*, 2002; Shen *et al.*, 2001; Yamamoto *et al.*, 2008; Yoshida *et al.*, 2003, 2001).

In contrast, the mechanism of the Golgi stress response is less clear, and only three response pathways have been reported (Taniguchi and Yoshida, 2017). The first identified pathway of the mammalian Golgi stress response was the TFE3 pathway, which regulates expression of N-glycosylation enzymes, Golgi structural proteins and vesicular transport components. The transcription factor and the enhancer regulating the TFE3 pathway are TFE3 (Taniguchi *et al.*, 2015) and GASE (Oku *et al.*, 2011), but the sensor of the TFE3 pathway has not been identified. In normal growth conditions, TFE3 is phosphorylated at serine 108 (Ser108) and retained in the cytosol as a dormant form, whereas TFE3 is dephosphorylated and translocated to the nucleus upon Golgi stress, where it activates transcription of Golgi-related genes by binding GASE. MLX is a negative regulator of the TFE3 pathway and competitively inhibits the GASE-binding of TFE3 (Taniguchi *et al.*, 2016). In addition to the TFE3 pathway, Tohyama and colleagues reported the HSP47 pathway of the Golgi stress response, which activates expression of the ER chaperone HSP47 in order to prevent Golgi stress-induced apoptosis (Miyata *et al.*, 2013). Moreover, Reiling and colleagues identified the third pathway of the Golgi stress response, the CREB3 pathway, which activates the transcription factor CREB3 and induces apoptosis upon Golgi stress via transcriptional activation of ARF4 (Gendarme *et al.*, 2017; Ignashkova *et al.*, 2017; Ramirez-Peinado *et al.*, 2017; Reiling *et al.*, 2013; van Raam *et al.*, 2017) (see discussion).

Proteoglycans (PGs) are a kind of glycoprotein consisting of a PG core protein and several long chains of glycans referred to as glycosaminoglycans (Nadanaka and Kitagawa, 2018). PGs have several functions, including lubrication in the joints, water retention in the skin and negative regulation of neurite outgrowth by reactive astrocytes in neural tissues (Sakamoto and Kadomatsu, 2017). As glycans of PGs, which are conjugated to PG core proteins in the Golgi, are critical for the function of PGs described above, glycosylation of PGs is one of the important functions of the Golgi. It is highly possible that synthesis of PG core proteins is increased during differentiation of PG-producing cells, such as chondrocytes, skin fibroblasts and reactive astrocytes, and overwhelms the capacity of glycosylation for PGs in the Golgi (PG-Golgi stress). Upon such situations, cells likely activate the Golgi stress response in order to upregulate expression of glycosylation enzymes for PGs. However, the TFE3, HSP47 and CREB3 pathways are unable to regulate expression of glycosylation enzymes for PGs, suggesting that expression of glycosylation enzymes for PGs is controlled by an unidentified Golgi stress response pathway. We named this pathway the PG pathway, and have been investigating its molecular mechanism. In this report, we present target genes of the PG pathway, as well as the enhancer element PGSE (PG stress response element) that regulates transcriptional induction of glycosylation enzymes for PGs in response to PG-Golgi stress. Our study clarified the existence of the PG pathway and the basics of its molecular mechanism.

## Materials and Methods

### Cell culture and transfection

HeLa cells were cultured in Dulbecco’s modified Eagle’s medium (glucose at 4.5 g/liter) supplemented with 10% fetal calf serum, 2 mM glutamine, 100 units/ml of penicillin and 100 g/ml of streptomycin at 37°C in a humidified 5% CO_2_, 95% air atmosphere (Yoshida *et al.*, 2006). When cells were transfected with plasmid DNA, the calcium phosphate method was used, whereas siRNAs were transfected into cells using the Lipofectamine RNAiMAX transfection reagent (Thermo-Fischer Scientific, Waltham, MA) (Komori *et al.*, 2012). After transfection, cells were treated with Golgi stress inducers for 16–18 h, washed three times with PBS, and then harvested for immunoblotting, immunocytochemistry and luciferase assays (Uemura *et al.*, 2013). The nuclear and cytoplasmic fractions were separated by centrifugation after dissolving the plasma membrane with NP40 (Schreiber *et al.*, 1989) (Uemura *et al.*, 2009).

### Construction of plasmids and transfection of siRNAs

The expression plasmid for human SDC2 was kindly provided by Drs. Hiroshi Kitagawa and Satomi Nadanaka (Nadanaka and Kitagawa, manuscript in preparation). The plasmid expressing human TFE3 was constructed in the previous paper (Taniguchi *et al.*, 2015). For construction of promoter-luciferase reporter vectors, corresponding promoter regions of human GLCE, HS6ST1 and NDST2 were amplified from human genomic DNA, and cloned into the BglII site of the GL4 basic vector (Promega, Fitchburg, WI). Point mutants of the vectors were constructed by site-directed mutagenesis using a QuikChange Site-Directed Mutagenesis kit (Stratagene, CA). The human BiP-luciferase construct and its mutant were constructed previously (Yoshida *et al.*, 1998). The nucleotide sequences of siRNAs used in this study were as follows: siTFE3-A (CAGAAGAAAGACAAUCACAACCUAA), siTFE3-B (GGGAAUCUGCUUGAUGUGUACAGUA), siSp1-A (GCAACAUGGGAAUUAUGAATT), siSp1-B (GGCAGACCUUUACAACUCATT) and siSp1-C (CCACAAGCCCAAACAAUCATT). Silencer Select Negative Control #4390843 (Life Technologies, CA) was used as the control siRNA. Transfection of siRNA was carried out using the lipofection agent RNAi MAX (Thermo-Fischer Scientific), and transfected cells were incubated for 48 h after transfection.

### Immunoblotting and immunocytochemistry

Immunoblotting and immunocytochemistry were carried out as described previously (Yoshida *et al.*, 2009). Anti-ATF6, anti-XBP1, anti-Giantin and anti-GM130 antisera were purchased from Cosmo Bio (Tokyo, Japan) (clone 1–7), Santa Cruz Biotechnology (TX) (sc-7160), COVANCE (Princeton, NJ) (PRB-114C) and Becton, Dickinson and Company (Franklin Lakes, New Jersey) (610823), respectively. Anti -TFE3 antiserum was prepared in the previous study (Taniguchi *et al.*, 2015).

### Quantitative RT-PCR and luciferase assays

Quantitative RT-PCR (qRT-PCR) was performed using a QuantStudio 6 Flex Real-Time PCR System (Thermo-Fischer Scientific) and a PrimeScript RT reagent kit with gDNA Eraser and SYBR Premix Ex Taq II (Tli RNase H Plus) (TaKaRa, Otsu, Japan). Primer pairs used for qRT-PCR were as follows: *NDST2* (CCTGTGATGACAAGAGGCACAAA and TGCAGGCTCAGGAAGAAGTGAAT), *HS6ST1* (TCACCTTCACCATGGGCTTC and GACTGAGACAAGACCCGTGCTTC), *GLCE* (GGCTACAATGTGGAAGTCCGAGA and CTGGATTGGATAGAAATAGCCTTGA), *CSGALNACT2* (CGTGGACGAGGACTAAATGTGG and GTTGGCATAAACAATGGCAGGA), *B3GAT3* (GAGCAGTCTTCTGAGCCACCTTG and CTGCTTCATCTTGGGCTTCTCTG), *ARF4* (GAGATAGTCACCACCATTCCTACCA and GGCCTAATTCTATCTTGACCACCA), *SERPINH1* (AAGAGCAGCTGAAGATCTGGATG and GTCGGCCTTGTTCTTGTCAATG), *TFE3* (ACTGGGCACTCTCATCCCTAAGTC and TTCAGGATGGTGCCCTTGTTC), *Sp1* (TCACACGTTCGGATGAGCTACAG and ATGAAGCGCTTAGGACACTCAGG) and *PCYT1A* (ATGTGTATGCGAGGCGGAAC and CCACTTCTGAATGAGGTCAATGCT). Assays for firefly luciferase were carried out with the PicaGene Dual Sea Pansy luminescence kit (Toyo Ink, Tokyo, Japan) (Yoshida *et al.*, 2006).

### Microarray analyses and RNA-Seq with next generation DNA sequencer

Total RNA prepared from HeLa cells treated with 7.5 mM 4MU-xyloside for 16 h was purified with the RNeasy Mini Kit (Qiagen), and subjected to microarray and RNA-seq analyses. Microarray analyses were performed using the SurePrint G3 Human GE 8x60K v2 Microarray (Agilent Technologies, Santa Clara, CA) in collaboration with TaKaRa Bio Inc. RNA-Seq analyses were performed with the Ion Proton System for Next-Generation Sequencing with PI chips (Thermo Fisher Scientific, Waltham, MA). The microarray and RNA seq data were deposited in GEO database (GEO accession numbers are GSE117938 and GSE119909, respectively).

## Results

### Insufficiency of PG glycosylation resulted in fragmentation of the Golgi

Currently, there are no known experimental methods to induce PG-Golgi stress *in vitro*. For ER stress, two methods are usually employed: (1) overexpression of secretory or membrane proteins and (2) inhibition of ER function, such as protein folding in the ER, by chemicals. Overexpression of secretory proteins overwhelms the capacity of ER chaperones, resulting in insufficient protein folding capacity (ER stress) and accumulation of unfolded proteins in the ER. Inhibition of protein folding by chemicals, such as tunicamycin, thapsigargin or dithiothreitol, also results in accumulation of unfolded proteins in the ER, which is recognized as insufficiency of folding capacity by cells (Wakabayashi and Yoshida, 2013). Thus, to evoke PG-Golgi stress, we tested two treatments that reduce the capacity for PG glycosylation in the Golgi: overexpression of the PG core protein SDC2 and inhibition of PG glycosylation by the chemical inhibitors 4MU-xyloside and 4NP-xyloside.

**Fig. 1:**
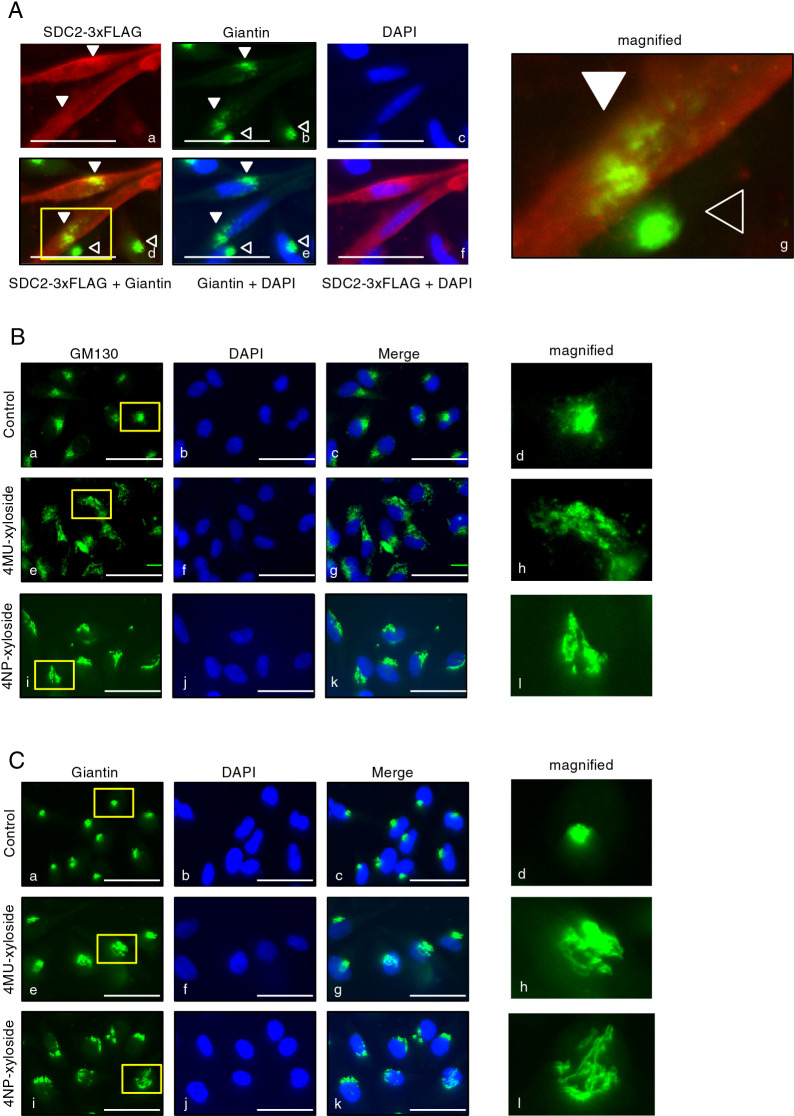
Morphological changes in the Golgi apparatus caused by PG-Golgi stress. (A) HeLa cells transfected with SDC2-3xFLAG were stained with anti-FLAG (red), anti-Giantin (green) antisera and DAPI (blue). Each panel shows staining for anti-FLAG (panel a), anti-Giantin (panel b), DAPI (panel c), anti-FLAG and anti-Giantin (panel d), anti-Giantin and DAPI (panel e), and anti-FLAG and DAPI (panel f). Right panels show magnified images of left panels indicated by white rectangle. Bars=50 μm. (B and C) HeLa cells treated with 7.5 mM 4MU-xyloside for 16 h or 6 mM 4NP-xyloside for 18 h were stained with anti-GM130 (green in (B)) or anti-Giantin (green in (C)) antiserum and DAPI (blue), respectively. Right panels show magnified images of left panels indicated by white rectangle. Bars=50 μm.

SDC2 is a well-known core protein of PGs (Essner *et al.*, 2006), and overexpression of SDC2 may exceed the capacity of glycosylation enzymes for PGs and cause insufficient PG glycosylation (PG-Golgi stress). The human SDC2 gene was tagged with three copies of the FLAG tag (SDC2-3xFLAG) and transfected into HeLa cells, which were stained with anti-Giantin (a cis- and medial-Golgi marker) and anti-FLAG antisera (Fig. 1A). In HeLa cells that did not overexpress SDC2 (open arrowheads), the Golgi was compact and localized near the nucleus, whereas the Golgi in SDC2-overexpressing cells (closed arrowheads) was expanded and fragmented (panel b, d, e and g). Of note, a considerable proportion of SDC2 was accumulated in the Golgi apparatus (closed arrowheads) (panels a and d). The abnormal Golgi morphology and SDC2 accumulation supported that overexpression of SDC2 induced PG-Golgi stress.

Next, we examined whether chemical inhibitors of PG glycosylation, 4MU-xyloside and 4NP-xyloside, affect Golgi morphology. 4MU-xyloside and 4NP-xyloside are analogs of xylose, and competitively inhibit glycosylation of PG core proteins because xylose is the first sugar conjugated to PG core proteins. Other sugars are conjugated linearly, and xylosides can accept these sugars (Kanwar *et al.*, 1984; Schwartz, 1977). It was reported that 4NP-xyloside treatment causes accumulation of PGs in the Golgi and altered Golgi morphology (Kanwar *et al.*, 1986). When HeLa cells were treated with either 4MU-xyloside or 4NP-xyloside and stained with anti-GM130 (a cis-Golgi marker) or anti-Giantin antiserum, the Golgi was highly fragmented (Fig. 1B and 1C, panels e–l), suggesting that xyloside treatment induces PG-Golgi stress. From these observations, we speculated that SDC2 overexpression and xylosides can be used to evoke PG-Golgi stress.

To reveal whether xyloside treatment affects other organelles, HeLa cells treated with 4MU-xyloside or 4NP-xyloside were stained with an ER marker (calnexin), a mitochondria marker (COX IV) and a lysosome marker (LAMP1) (Fig. S1A–C). We found that morphology of these organelles was less affected by xyloside treatment, while cell shape becomes slightly narrow (Fig. S1D).

### PG-Golgi stress induces transcription of PG-related genes

**Fig. 2:**
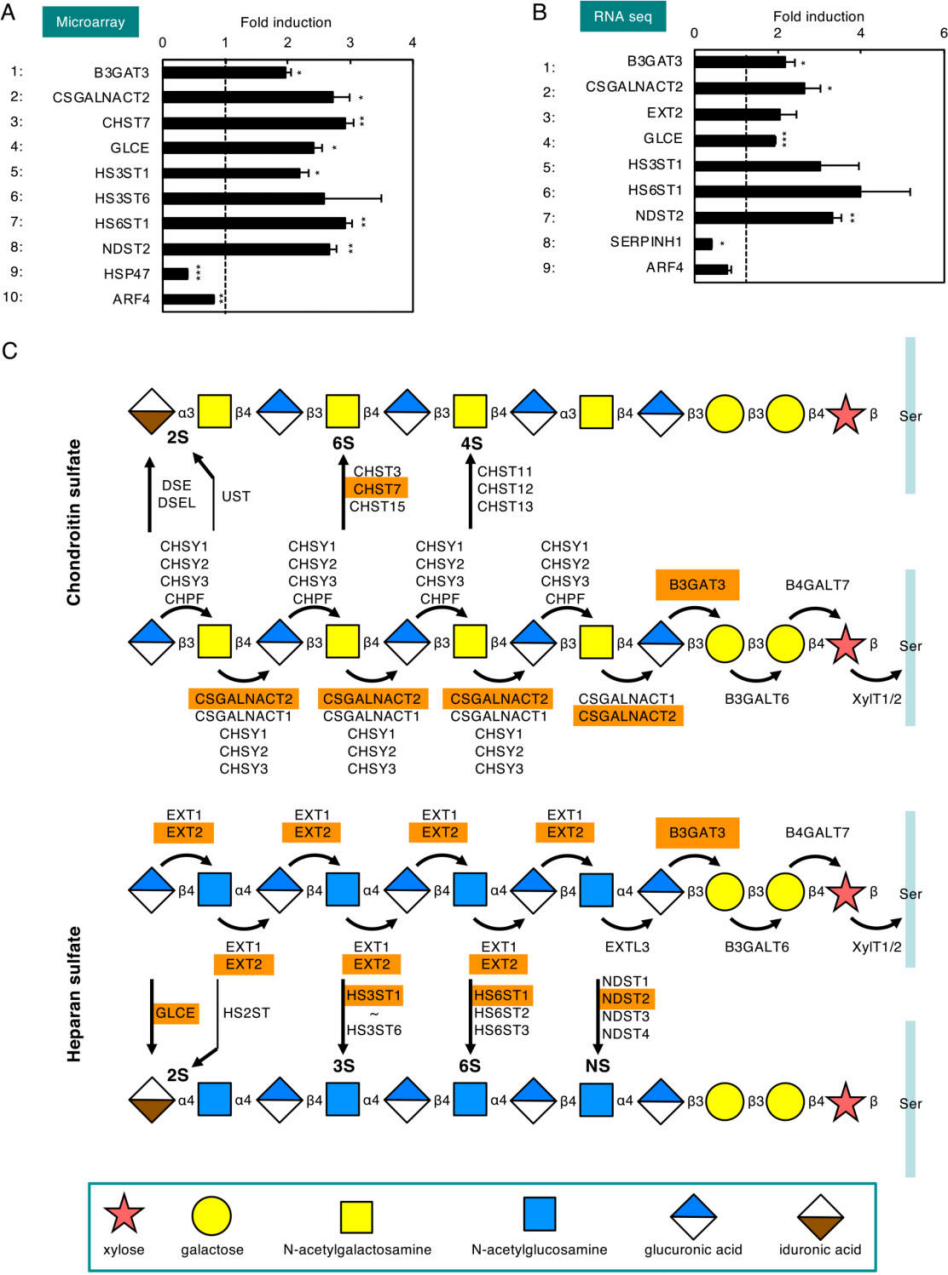
Screening of PG-related genes whose expression is induced by xyloside treatment. (A and B) Total RNA prepared from HeLa cells treated with 7.5 mM 4MU-xyloside for 16h was subjected to (A) microarray and (B) RNA seq analyses. Data for PG-related genes whose expression was induced more than 1.9-fold in 4MU-xyloside treated cells as compared with untreated cells are shown. Target genes of the HSP47 and CREB3 pathways (HSP47 and ARF4) are also shown. Values are means±SE of three independent experiments. ***, P<0.001; **, P<0.01; *, P<0.05. (C) Glycosylation pathways of chondroitin sulfate proteoglycan (upper panel) and heparan sulfate proteoglycan (lower panel), and their responsible enzymes. Enzymes whose expression was induced by xyloside in (A) and (B) are indicated in orange. Sugars are conjugated to the serine residue of a core protein one by one. Some sugars are sulfated (indicated by 2S, 3S, 4S, 6S and NS), and some glucuronic acids are epimerized to iduronic acid.

**Fig. 3:**
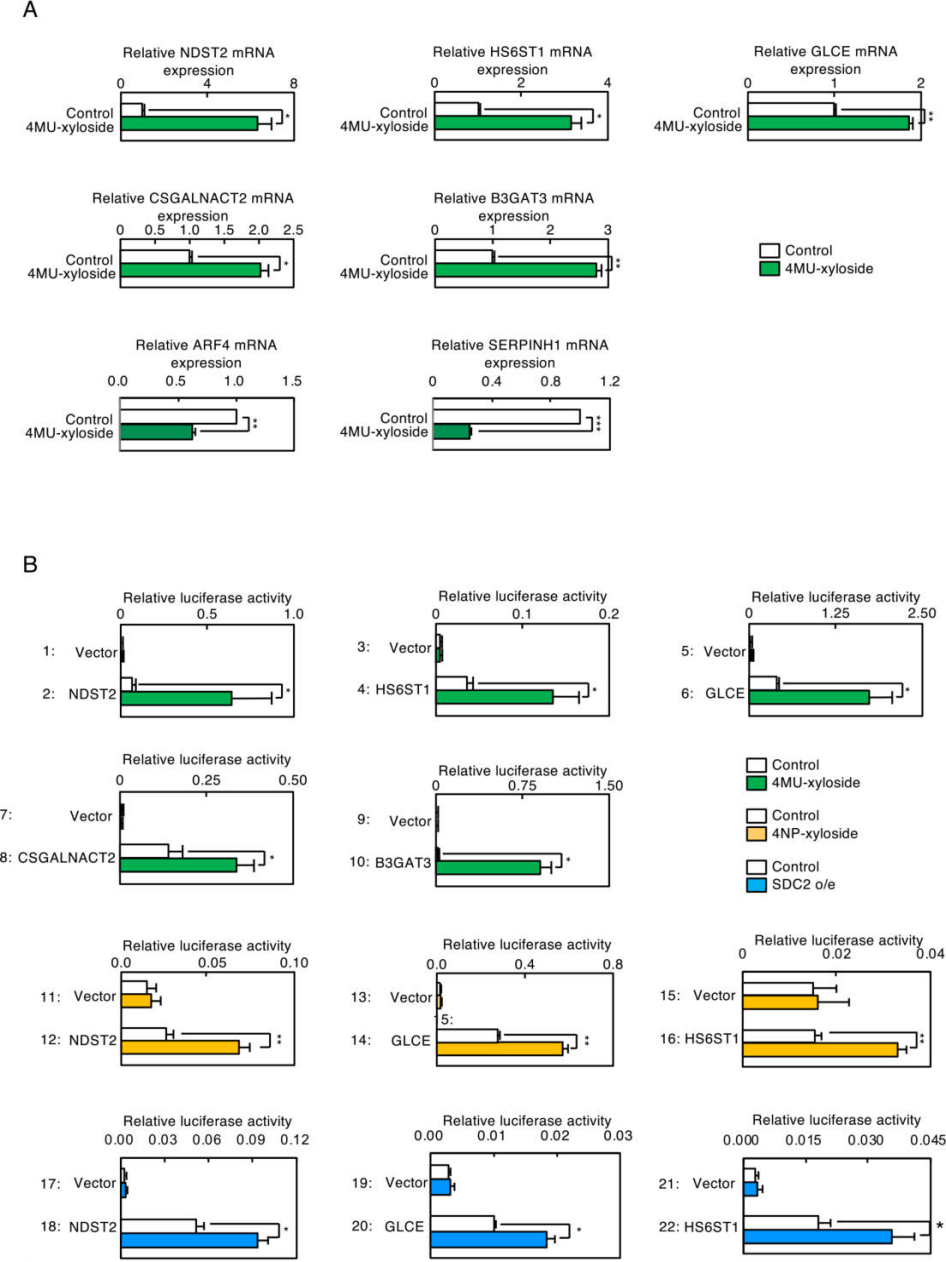
Effects of PG-Golgi stress on expression of PG-related genes. (A) Total RNA prepared from HeLa cells treated with 7.5 mM 4MU-xyloside for 16 h was subjected to qRT-PCR experiments to measure mRNA levels of indicated PG-related genes. Expression of ARF4 and HSP47 were also evaluated. Values are means±SE of three independent experiments. ***, P<0.001; **, P<0.01; *, P<0.05. (B) HeLa cells transfected with a luciferase reporter gene fused with indicated human promoters of PG-related genes were treated with 7.5 mM 4MU-xyloside (lanes 1–10), 6 mM 4NP-xyloside for 18 h (lanes 11–16) or co-transfected with an SDC2 expression vector (lanes 17–22), and subjected to the luciferase assay. Values are means±SE of three independent experiments. ***, P<0.001; **, P<0.01; *, P<0.05.

To examine whether xyloside treatment induces transcription of genes encoding PG glycosylation enzymes, RNA was prepared from HeLa cells treated with 4MU-xyloside and subjected to microarray analyses (Fig. 2A). We found that expression of PG-related genes was upregulated upon 4MU-xyloside treatment (lanes 1–8). B3GAT3 is a glycan elongation enzyme for chondroitin sulfate PGs (CSPGs) and heparan sulfate PGs (HSPGs) (Fig. 2C). CSGALNACT2 is a glycan elongation enzyme for CSPGs, and CHST7 is a glycan sulfation enzyme for CSPGs. GLCE, HS3ST1, HS3ST6, HS6ST1 and NDST2 are glycan isomerization or sulfation enzymes for HSPGs. RNA sequencing analyses were also performed using the same RNA samples, and we obtained results similar with those from the microarray analyses (Fig. 2B). EXT2 is a glycan elongation enzyme for HSPGs (Fig. 2C). To confirm that expression of NDST2, HS6ST1, GLCE, CSGALNACT2 and B3GAT3 was upregulated upon 4MU-treatment, we performed quantitative Real Time PCR (qRT-PCR) analyses for these genes (Fig. 3A), and found that these mRNAs were increased upon 4MU-xyloside treatment.

To examine whether induced expression of these genes is regulated at the level of transcription, promoter regions of these genes were isolated, fused with the firefly luciferase gene and transfected into HeLa cells (Fig. 3B). Transcription from the promoters can be evaluated by measuring the activity of luciferase. Transcription of the luciferase reporter gene fused with the human NDST2 promoter was markedly increased upon 4MU-xyloside treatment (lane 2), whereas that of the control construct was not affected by 4MU-xyloside treatment (lane 1). Similar results were obtained for the GLCE, HS6ST1, CSGALNACT2 and B3GAT3 promoters (lanes 3–10), and with 4NP-xyloside treatment (lanes 11–16). Of note, when SDC2 was overexpressed in HeLa cells, transcription from the promoters of NDST2, HS6ST1 and GLCE was significantly increased (lanes 17–22). These results indicate that PG-Golgi stress induces transcription of PG glycosylation genes.

### PG-Golgi stress-induced transcription is independent of TFE3, HSP47 and CREB3 Golgi stress response pathways

**Fig. 4:**
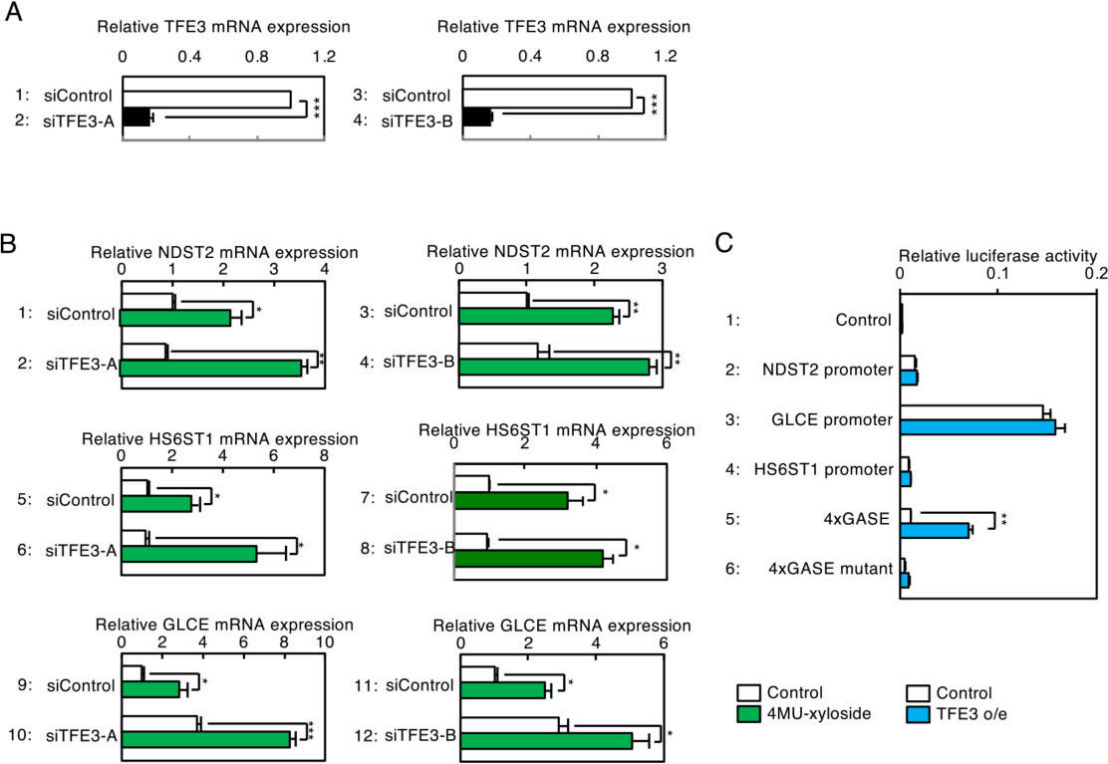
Effects of TFE3 knockdown or overexpression on expression of PG-related genes. (A) HeLa cells were transfected with siRNAs for TFE3 (siTFE3-A or siTFE3-B), and treated with 7.5 mM 4MU-xyloside for 16 h. Total RNA prepared from cells was subjected to qRT-PCR experiments, as in Fig. 3A. Values are means±SE of three independent experiments. ***, P<0.001; **, P<0.01; *, P<0.05. (B) HeLa cells were co-transfected with an indicated luciferase reporter as well as a TFE3 expression vector, and subjected to the luciferase assay. Values are means±SE of three independent experiments. ***, P<0.001; **, P<0.01; *, P<0.05.

Previously, we reported that TFE3 is dephosphorylated and translocates to the nucleus upon 4MU-xyloside treatment (Taniguchi *et al.*, 2015), suggesting that it is possible that the TFE3 pathway regulates transcriptional induction in response to xylosides. To examine whether transcriptional induction of the genes identified above is independent of the TFE3 pathway, we evaluated their induction in HeLa cells in which TFE3 expression was knocked-down. Expression of TFE3 was significantly suppressed by two different siRNAs for TFE3 (siTFE3-A and siTFE3-B) (Fig. 4A). When RNA prepared from cells treated with control siRNA (siControl) was subjected to qRT-PCR, expression of NDST2 mRNA was increased upon 4MU-xyloside treatment (lane 1). On the contrary, even if expression of TFE3 was knocked-down with siRNA (siTFE3-A), induction of NDST2 expression was instead enhanced (lane 2), indicating that the TFE3 pathway is not essential for transcriptional induction of the human NDST2 gene upon xyloside treatment. Similar results were obtained when different siRNA for TFE3 (siTFE3-B) was used (lanes 3 and 4). We obtained essentially the same results for HS6ST1 and GLCE (lanes 5–12), suggesting that xyloside-induced transcription of PG glycosylation genes is independent of the TFE3 pathway.

To confirm this, we examined whether expression of these genes is affected by overexpression of TFE3 (Fig. 4C). When GASE, an enhancer element that binds TFE3 and regulates transcriptional induction of the TFE3 pathway, was fused with the firefly luciferase gene and introduced into HeLa cells, transcription from GASE was markedly increased by TFE3 overexpression (lane 5), whereas that from mutant GASE was not (lane 6). In contrast, transcription from the NDST2, GLCE and HS6ST1 promoters was not affected by TFE3 overexpression (lanes 1–4). These observations demonstrate that transcriptional induction of NDST2, GLCE and HS6ST1 by xyloside is independent of the TFE3 pathway.

Next we investigated whether xyloside-induced transcription depends on the HSP47 or CREB3 pathway. HSP47 and ARF4 are well-known target genes of the HSP47 and CREB3 pathways, respectively (Miyata *et al.*, 2013; Reiling *et al.*, 2013). When HeLa cells were treated with 4MU-xyloside and subjected to microarray (Fig. 2A, lanes 9 and 10), RNA sequencing (Fig. 2B, lanes 8 and 9) and qRT-PCR analyses (Fig. 3A), expression of HSP47 and ARF4 mRNA was not induced but rather reduced, suggesting that the HSP47 and CREB3 pathways are not activated by xylosides. To examine whether CREB3 is activated by proteolysis upon xyloside treatment, we performed immunoblotting with whole cell extracts of HeLa cells treated with xylosides (Fig. S2). Since full length CREB3 was not detected in normally growing cells (lane 1), cells were treated with MG132, a proteasome inhibitor (lane 2). When cells were treated with brefeldin A (BFA), an authentic activator of the CREB3 pathway, full length CREB3 was reduced and cleaved (activated) CREB3 increased (lanes 3 and 4). In contrast, cleaved CREB3 was hardly detected upon treatment of 4MU- and 4NP-xylosides (lanes 5–11). From these results, we concluded that the HSP47 and CREB3 pathways cannot be activated by xyloside treatment, and that transcriptional induction of NDST2, GLCE, HS6ST1, CSGALNACT2 and B3GAT3 by xyloside is regulated by a novel response pathway separate from the TFE3, HSP47 and CREB3 pathways, which we named the PG pathway.

### Xyloside-induced transcription is independent of the ER stress response

**Fig. 5:**
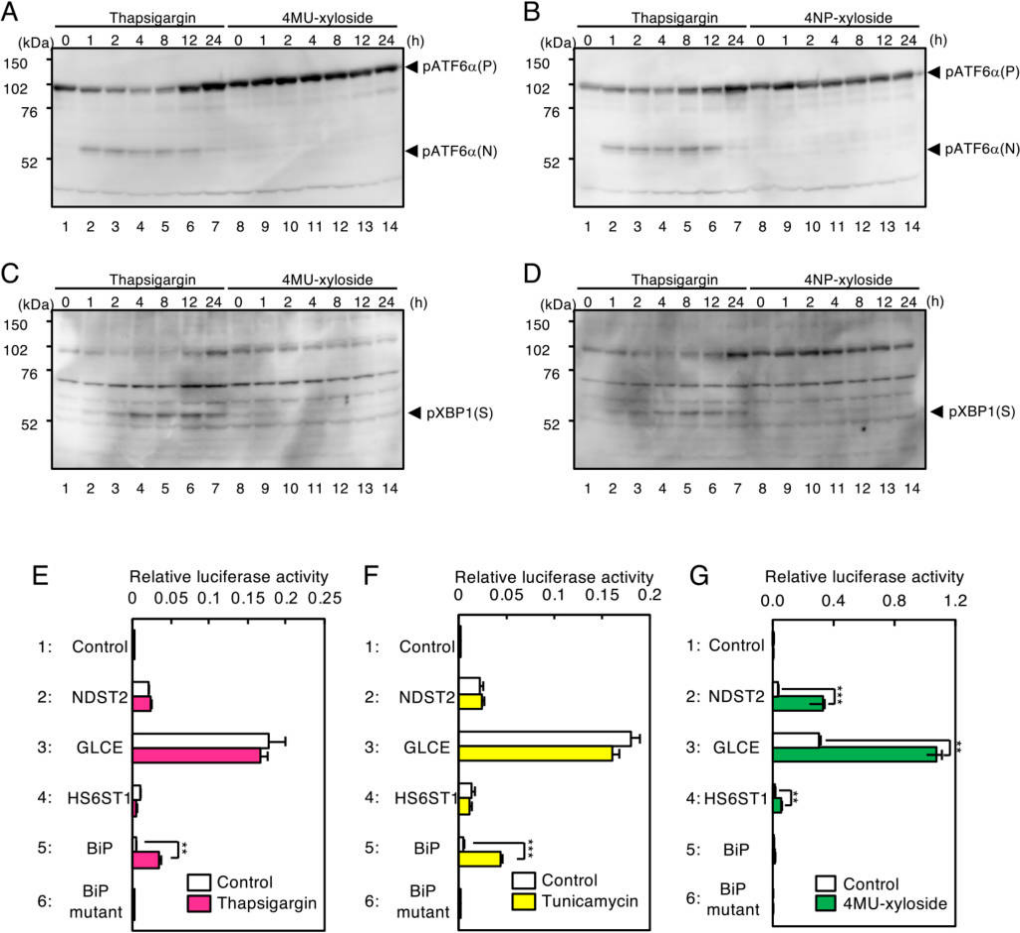
Effects of Golgi and ER stress inducers on ER and Golgi stress markers. (A–H) HeLa cells were treated with 1 μM thapsigargin, 7.5 mM 4MU-xyloside or 6 mM 4NP-xyloside for the indicated time, and subjected to immunoblotting with anti-ATF6α (A and B), and anti-XBP1 antiserum (C and D). Values are means±SE of three independent experiments. ***, P<0.001; **, P<0.01; *, P<0.05. (I–K) HeLa cells transfected with the indicated luciferase reporter were treated with (E) 300 nM thapsigargin for 16 h, (F) 6 μg/ml of tunicamycin for 16 h or (G) 7.5 mM 4MU-xyloside for 18 h, and subjected to the luciferase assay. Values are means±SE of three independent experiments. ***, P<0.001; **, P<0.01; *, P<0.05.

Next, we evaluated whether transcriptional induction of the PG-related genes by xyloside was independent of the ER stress response (Fig. 5). Thapsigargin is a well-known ER stress inducer that increases unfolded proteins in the ER by inhibiting Ca^2+^-ATPase in the ER (SERCA2) because Ca^2+^ is indispensable for activity of ER chaperones such as BiP and calreticulin. ATF6 is a sensor and transcription factor that regulates the ATF6 pathway, and mammalian cells have two ATF6 isoforms, ATF6α and ATF6β. In the absence of ER stress, pATF6(P)α is a transmembrane protein located in the ER membrane as a sensor molecule, whereas upon ER stress, pATF6(P)α is cleaved by proteolysis into cytosolic pATF6(N)α and luminal pATF6(L)α. pATF6(N)α liberated from the membrane translocates into the nucleus and activates transcription of ER chaperone genes. Thus, the cleavage status of ATF6 can be used as a marker of ER stress. As shown in Fig. 5A and 5B, only pATF6(P)α was observed in control cells (lane 1), whereas pATF6(N)α appeared upon thapsigargin treatment (lanes 2–7). Luminal pATF6(L)α cannot be detected by the anti-serum used in this experiment because it recognizes only the N-terminal part of pATF6(P)α corresponding to pATF6(N)α. In contrast, pATF6(N)α was not observed upon treatment with 4MU-xyloside or 4NP-xyloside (lanes 9–14), indicating that xyloside treatment does not activate ATF6. XBP1 is another ER stress marker whose pre-mRNA is converted to mature mRNA by unconventional mRNA splicing in response to ER stress, resulting in the production of pXBP1(S). Without thapsigargin treatment, pXBP1(S) was absent (Fig. 5C and 5D, lane 1), whereas pXBP1(S) was observed upon thapsigargin treatment (lanes 2–7). However, pXBP1(S) expression was not induced by 4MU- or 4NP-xyloside (lanes 9–14), demonstrating that XBP1 cannot be activated by xylosides. These observations strongly suggest that transcriptional induction by xylosides is independent of the ATF6 and IRE1 pathway of the ER stress response. As for the PERK pathway of the ER stress response, we investigated later (Fig. S3: see below).

Lastly, we confirmed these results by reporter assay using the promoters of PG-related genes and the ER chaperone gene BiP (Fig. 5E–G). When HeLa cells transfected with a luciferase reporter gene fused with the human BiP promoter were treated with thapsigargin, luciferase activity markedly increased (Fig. 5E, lane 5), whereas it was not upregulated with the mutant BiP promoter (lane 6). On the other hand, luciferase activity was unaffected by thapsigargin treatment in the case of NDST2, HS6ST1 and GLCE promoters (lanes 2–4). The same results were obtained when HeLa cells were treated with tunicamycin, another ER stress inducer (Fig. 5F). In contrast, when HeLa cells were treated with 4MU-xyloside, luciferase activity was not increased using the BiP promoter-luciferase construct (Fig. 5G, lane 5), whereas it was increased using the constructs containing promoters of PG-related genes (lanes 2–4). From these results, we concluded that xylosides activate transcription of PG-related genes not by the ER stress response but by the PG pathway of the Golgi stress response.

### Identification of an enhancer element regulating the PG pathway

**Fig. 6:**
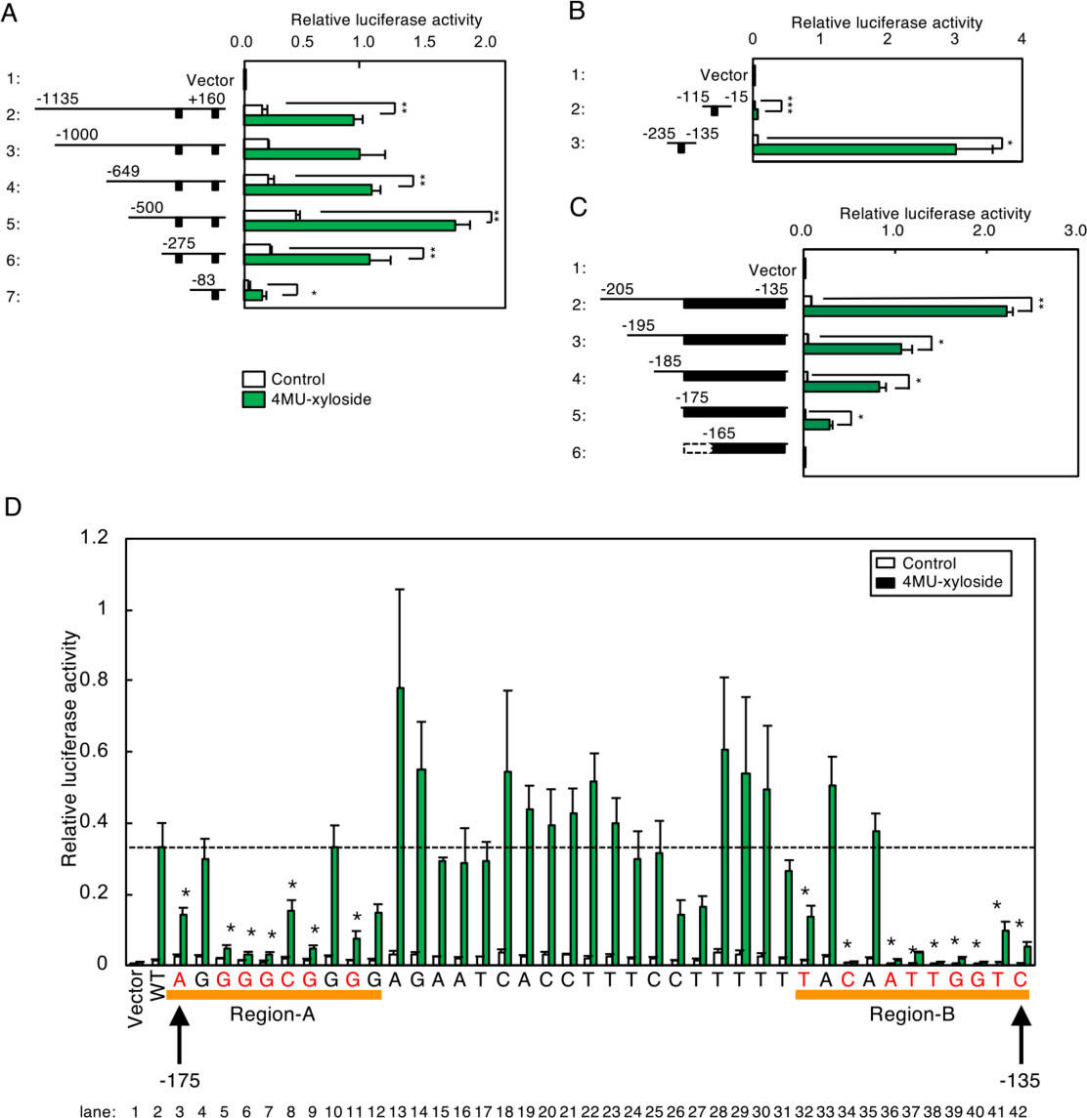
Systematic mutation analysis of the human GLCE promoter. (A–C) HeLa cells were transfected with reporter constructs containing the indicated regions of the human GLCE promoter and luciferase gene, treated with 7.5 mM 4MU-xyloside for 18 h and subjected to the luciferase assay. The estimated locations of enhancers regulating transcriptional induction are indicated by black boxes. Values are means±SE of three independent experiments. ***, P<0.001; **, P<0.01; *, P<0.05. (D) HeLa cells were co-transfected with a luciferase reporter containing the [–175 to –135] region as well as an SDC2 expression vector, and subjected to the luciferase assay. Values are means±SE of four independent experiments. ***, P<0.001; **, P<0.01; *, P<0.05. (E) Each single nucleotide in the [–175 to –135] region was replaced by another nucleotide (A, T, G and C were replaced by C, G, T and A, respectively), and its transcriptional induction activity upon treatment with 7.5 mM 4MU-xyloside for 18 h was evaluated. Point mutations that significantly reduced transcriptional induction upon xyloside treatment are marked by asterisks. Values are means±SE of three independent experiments. ***, P<0.001; **, P<0.01; *, P<0.05.

We next examined enhancer elements regulating transcriptional induction of human GLCE, NDST2, HS6ST1 and B3GAT3 genes in response to PG-Golgi stress. As the [–1135 to +160] region of the human GLCE promoter (numbers indicate the position from the transcription start site) increased transcription upon 4MU-xyloside treatment (Fig. 3B), we made a set of promotor deletion constructs fused with the firefly luciferase gene, and introduced them into HeLa cells to evaluate their activity for transcriptional induction in response to 4MU-xyloside treatment (Fig. 6A). We found that the [–275 to +160] region exhibited strong transcriptional induction activity, similar with that by the [–1135 to +160] region (lanes 2 and 6), whereas the [–83 to +160] region exhibited weak but significant activity (lane 7), suggesting that the [–275 to +160] region has at least two separate enhancer elements. Indeed, the [–115 to +15] and [–235 to –135] regions demonstrated transcriptional induction activity (Fig. 6B, lanes 2 and 3). As the [–235 to –135] region exhibited stronger activity, we further analyzed this region, and found that the [–175 to –135] region had significant activity upon xyloside treatment (Fig. 6C, lane 5). We then introduced single point mutations for each nucleotide of the [–175 to –135] region, and evaluated the activity of each mutant (Fig. 6D). The wild type [–175 to –135] region exhibited strong transcriptional induction activity upon 4MU-xyloside treatment (lane 2), but point mutations around Region-A and Region-B significantly reduced the activity (lanes 3–12 and 32–42), suggesting that these regions are critical for transcriptional induction upon PG-Golgi stress.

**Fig. 7:**
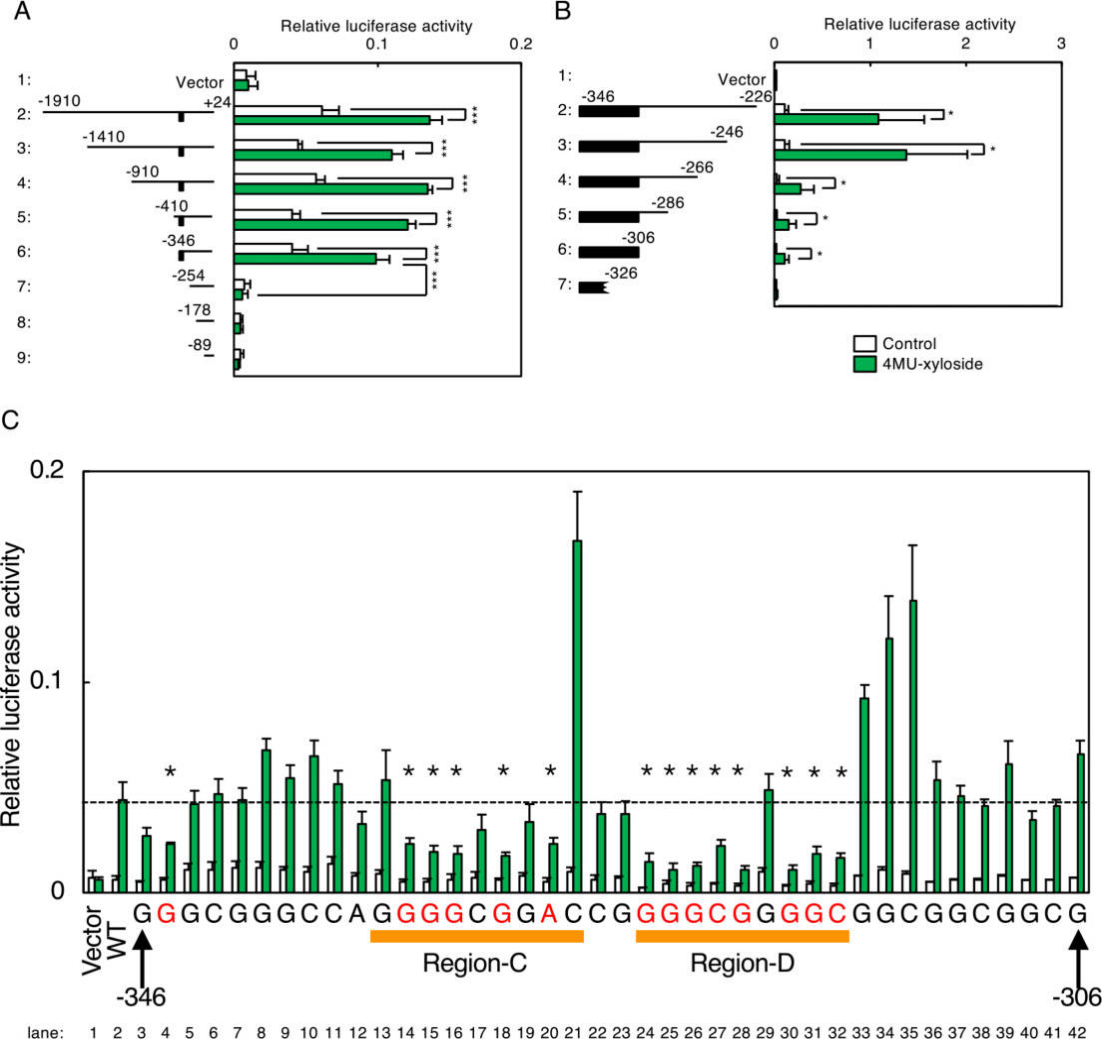
Systematic mutation analysis of the human HS6ST1 promoter. (A and B) Deletion analysis of the human HS6ST1 promoter was performed as described in Fig. 6A–C. Values are means±SE of three independent experiments. ***, P<0.001; **, P<0.01; *, P<0.05. (C) HeLa cells were co-transfected with a luciferase reporter containing two copies of the [–346 to –306] region as well as an SDC2 expression vector, and subjected to the luciferase assay. Values are means±SE of three independent experiments. ***, P<0.001; **, P<0.01; *, P<0.05. (D) Point mutation analysis of the [–346 to –306] region was performed as described in Fig. 6E. Values are means±SE of three independent experiments. ***, P<0.001; **, P<0.01; *, P<0.05.

The human HS6ST1 promoter was also similarly analyzed. The [–346 to +24] region demonstrated significant transcriptional induction activity, similar with the [–1910 to +24] region (Fig. 7A, lane 6), whereas the [–254 to +24] region did not (lane 7), suggesting that the region around –346 contains enhancer elements. Furthermore, the [–346 to –306] region exhibited significant activity in response to xyloside treatment (Fig. 7B, lane 6). We next introduced point mutations for each nucleotide in the [–346 to –306] region, and evaluated the effects of the mutations on transcriptional induction (Fig. 7C). Compared with wild type (lane 2), point mutations around Region-C and Region-D significantly reduced the activity (lanes 14–20 and 24–32).

**Fig. 8:**
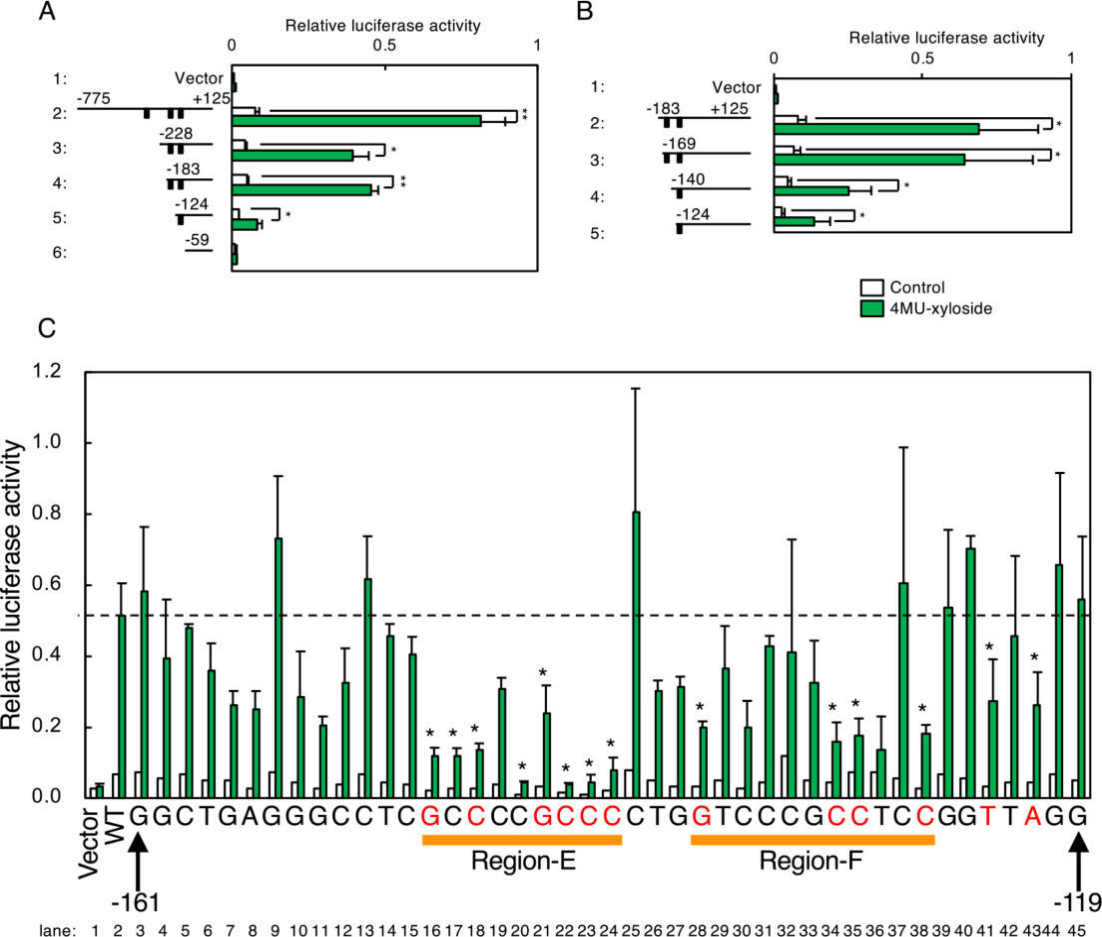
Systematic mutation analysis of the human NDST2 promoter. (A and B) Deletion analysis of the human NDST2 promoter was performed as described in Fig. 6A–C. Values are means±SE of three independent experiments. ***, P<0.001; **, P<0.01; *, P<0.05. (C) HeLa cells were co-transfected with a luciferase reporter containing the [–161 to –119] region as well as an SDC2 expression vector, and subjected to the luciferase assay. Values are means±SE of four independent experiments. ***, P<0.001; **, P<0.01; *, P<0.05. (D) Point mutation analysis of the human NDST2 promoter was performed as described in Fig. 6E. Values are means±SE of three independent experiments. ***, P<0.001; **, P<0.01; *, P<0.05.

Regarding the human NDST2 promoter, we constructed a reporter gene containing the luciferase gene fused with the [–775 to +125] region of the NDST2 promoter and its deletions (Fig. 8A) because there is another gene (CAMK2G) 776 nt upstream of the NDST2 gene. The [–775 to +125] region produced strong induction activity (lane 2), whereas deletion of the [–775 to –229] region resulted in reduced activity (lane 3), suggesting that the [–775 to –229] region contains an enhancer element responsible for transcriptional induction. Similarly, deletion of the [–183 to –125] region as well as the [–124 to –60] region reduced the activity (lane 5), suggesting existence of other enhancer elements in these regions. Deletion of the [–183 to –170] region hardly affected the activity (Fig. 8B, lane 3), whereas that of the [–169 to –141] region affected activity (lane 4), suggesting that the enhancer resides around the [–169 to –141] region. When the [–169 to –119] region was tested, transcriptional induction activity was noted upon xyloside treatment (Fig. 8C, lane 2). Point mutations were then introduced for each nucleotide in the [–169 to –119] region, and their effects on transcriptional induction were evaluated. We found that point mutations in the Region-E and Region-F reduced activity (lanes 16–24 and 28–38).

**Fig. 9:**
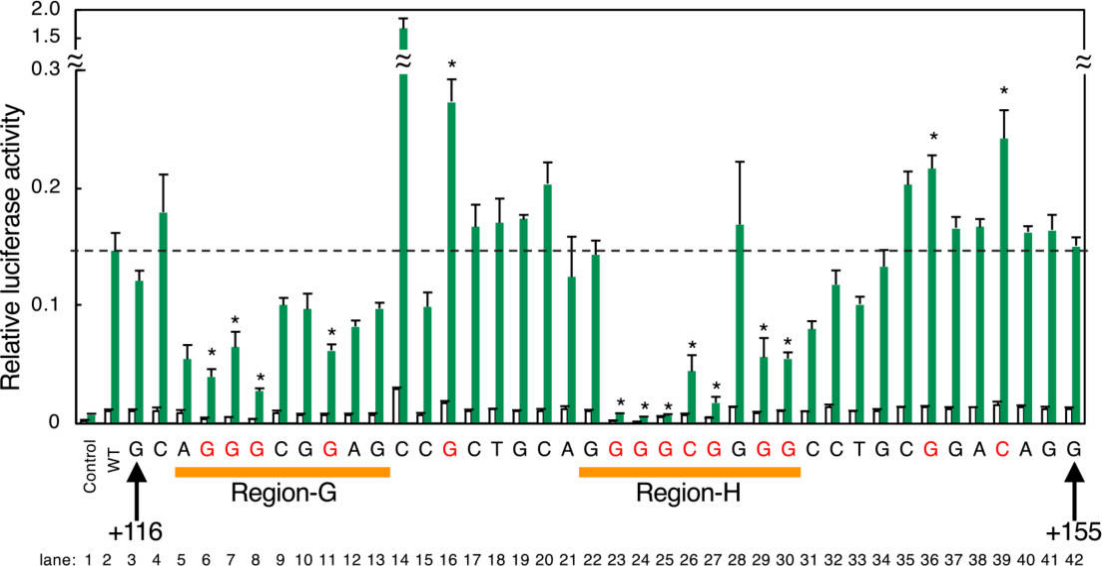
Point mutation analysis of the PGSE-like sequences in the human B3GAT3 promoter. Point mutation analysis of PGSE-like sequences found in the [+116 to +155] region of the human B3GAT3 promoter was performed as described in Fig. 6E. Values are means±SE of three independent experiments. ***, P<0.001; **, P<0.01; *, P<0.05.

We searched the human B3GAT3 promoter for nucleotide sequences similar to Region-A to F, and found that the [+116 to +155] region contains a similar nucleotide sequence, excluding Region-B. Transcription from the [+116 to +155] region was increased upon xyloside treatment (Fig. 9E, lane 2), and point mutation analysis revealed that Region-G and Region-H are critical for transcriptional induction.

**Fig. 10:**
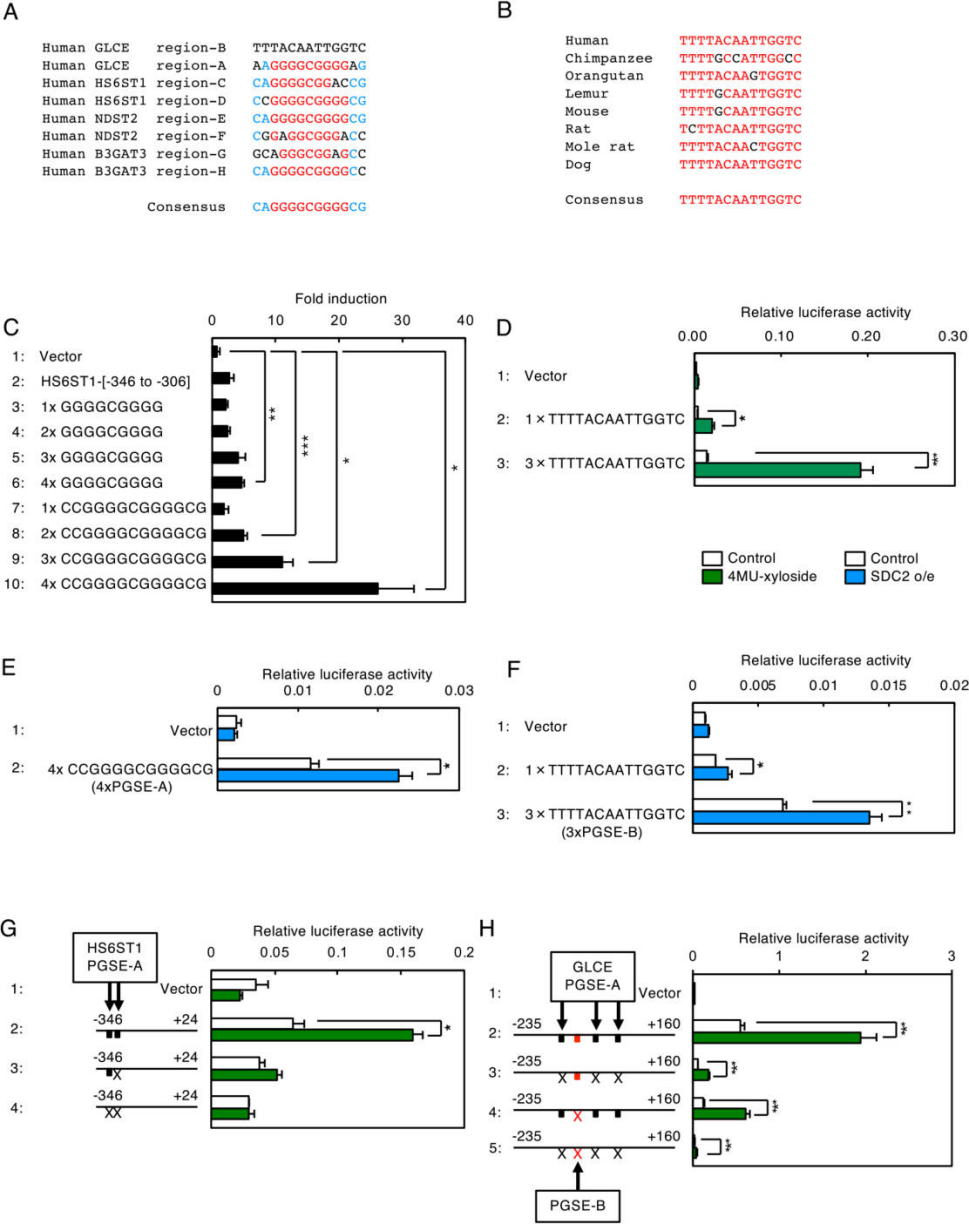
PGSE is necessary and sufficient for transcriptional induction upon PG-Golgi stress. (A) Comparison of nucleotide sequences in Regions A–H and their surrounding regions. The highly conserved nucleotides are highlighted in red and blue. (B) Comparison of nucleotide sequences in Region-B among mammals. (C) Transcriptional induction activity of Region-D and its surrounding sequences. HeLa cells were transfected with luciferase reporters fused with the indicated sequences, treated with 7.5 mM 4MU-xyloside for 18 h, and subjected to the luciferase assay. Values are means±SE of three independent experiments. ***, P<0.001; **, P<0.01; *, P<0.05. (D) Transcriptional induction activity of Region-B and its surrounding sequences was evaluated as described in (C). (E and F) Transcriptional induction activity of (E) Region-D and (F) Region B with their surrounding sequences upon SDC2 overexpression was evaluated as described in Fig. 8D. (G) HeLa cells transfected with the [–346 to +24] region of the human HS6ST1 promoter in which one or two PGSE-A sequences were disrupted were treated with 7.5 mM 4MU-xyloside for 18 h, and subjected to the luciferase assay. The location of wild-type and disrupted PGSE-A sequences are indicated by black boxes and X, respectively. Values are means±SE of three independent experiments. ***, P<0.001; **, P<0.01; *, P<0.05. (H) HeLa cells transfected with the [–235 to +160] region of the human GLCE promoter were analyzed as in (G). The locations of PGSE-A and PGSE-B sequences are indicated by black and red boxes, respectively.

When the nucleotide sequences of Regions-A to -H were compared, all of the nucleotide sequences except for those of Region-B were found to contain GGGGCGGGG-like sequences (Fig. 10A). However, tandem repeats of the GGGGCGGGG sequence did not increase transcription upon xyloside treatment (Fig. 10C, lanes 3–6). As several nucleotides outside of the GGGGCGGGG sequence are considerably conserved (Fig. 10A), we constructed tandem repeats of the CCGGGGCGGGGCG sequence and found that this sequence exhibited strong induction activity upon xyloside treatment (Fig. 10C, lanes 7–10), indicating that the CCGGGGCGGGGCG sequence is the consensus sequence for the enhancer element that sufficiently responds to PG-Golgi stress. We named this enhancer element PG-Golgi stress response element A (PGSE-A). As for Region-B, the nucleotide sequence around Region-B is well conserved among mammals with a consensus sequence of TTTTACAATTGGTC. When tandem repeats of TTTTACAATTGGTC were similarly tested, we found that it also supported transcriptional induction upon xyloside treatment (Fig. 10D, lane 3). Thus, we named this sequence PGSE-B. Of note, when SDC2 was overexpressed in HeLa cells, 4xPGSE-A and 3xPGSE-B significantly increased transcription (Fig. 10E, lane 2 and Fig. 10F, lane 3), confirming that both PGSE-A and PGSE-B respond to PG glycosylation insufficiency (PG-Golgi stress).

Lastly, we mutated PGSE sequences in the human HS6ST1 and GLCE promoters in order to evaluate their contribution to transcriptional induction during Golgi stress. The [–346 to +24] region of the human HS6ST1 promoter contains two PGSE-A sequences, and transcription from this region significantly increased upon 4MU-xyloside treatment (Fig. 10G, lane 2). When these PGSE-A sequences were mutated, transcription from the HS6ST1 promoter was hardly induced (lanes 3 and 4), demonstrating that PGSE-A is essential for transcriptional induction of the HS6ST1 gene in response to PG-Golgi stress. Similarly, the [–235 to +160] region of the human GLCE promoter contains three PGSE-A sequences and one PGSE-B sequence. Mutation of the three PGSE-A sequences or one PGSE-B sequence alone did not abolish transcriptional induction (Fig. 10H, lanes 3 and 4), whereas mutation of all four PGSE sequences nearly abolished the activity completely (lane 5), indicating that both PGSE-A and PGSE-B are essential for transcriptional induction of GLCE. From these observations, we concluded that both PGSE-A and PGSE-B are enhancer elements regulating the PG pathway.

**Fig. 11:**
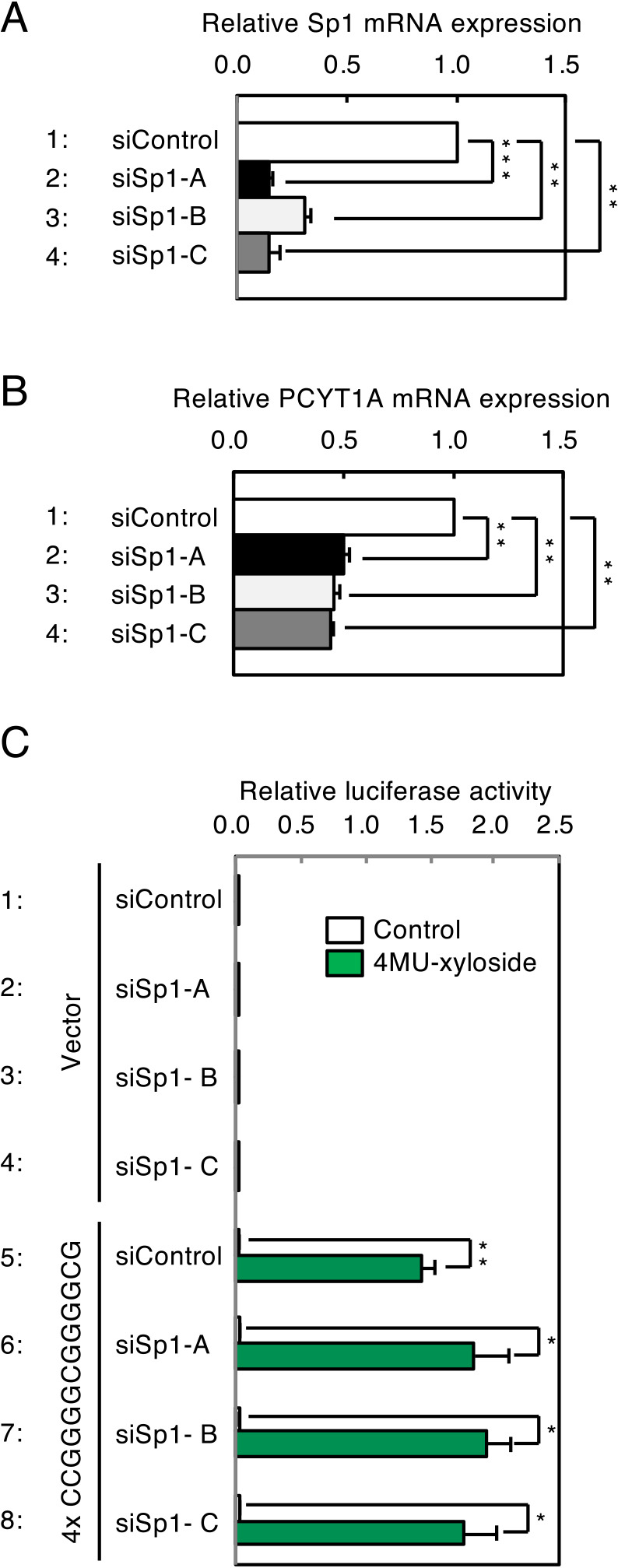
Effects of Sp1 knockdown on transcriptional induction of PG-related genes. (A) Evaluation of knockdown efficiency of Sp1 by qRT-PCR. Total RNA was prepared from HeLa cells transfected with siRNA for Sp1 (siSp1-A, siSp1-B and siSp1-C) and subjected to qRT-PCR. Values are means±SE of three independent experiments. ***, P<0.001; **, P<0.01; *, P<0.05. (B) Effects of Sp1 knockdown on the known Sp1 target gene, PCYT1A. (C) Effects of Sp1 knockdown on transcriptional induction by PGSE-A. HeLa cells transfected with a reporter plasmid containing 4x CCGGGGCGGGGCG and indicated siRNA were treated with 7.5 mM 4MU-xyloside for 18 h, and subjected to the luciferase assay. Values are means±SE of three independent experiments. ***, P<0.001; **, P<0.01; *, P<0.05.

The nucleotide sequence of PGSE-A includes the binding sequence of a well-known transcription factor, Sp1 (Archer, 2011). To examine whether Sp1 is involved in transcriptional induction through PGSE, we suppressed expression of Sp1 using siRNAs for Sp1 (siSp1-A, siSp1-B and siSp1-C). When transfected into HeLa cells, they suppressed expression of Sp1 mRNA (Fig. 11A, lanes 2–4), as compared with control siRNA (lane 1). PCYT1A, which encodes CTP:phosphocholine cytidylyltransferase α, is a well-known target gene of Sp1 (Banchio *et al.*, 2003). When expression of PCYT1A was measured by qRT-PCR, it was markedly reduced by transfection of siSp1s (Fig. 11B, lanes 2–4). In contrast, when HeLa cells were co-transfected with a 4xPGSE-A-LUC reporter and siSp1s, and treated with 4MU-xyloside, transcriptional induction from 4xPGSE-A was not affected by siSp1, suggesting that Sp1 is not essential for transcriptional induction from PGSE-A upon PG-Golgi stress.

Since all of above data were obtained only in HeLa cells, we investigated whether PGSEs are active in other cell lines, such as murine embryonic fibroblasts (MEFs) and human C6 glioma cells. These cells were transfected with either a PGSE-A or PGSE-B reporter vector and subjected to luciferase assays. As shown in Fig. S3, transcription from PGSE-A and PGSE-B was induced upon xyloside treatment in these cell lines (lanes 2 and 3), suggesting that our observations are not specific to HeLa cells but can be applied to various mammalian cells.

Using PGSE reporters, we finally investigated whether the PERK pathway of the ER stress response is involved in the transcriptional induction from PGSEs (Fig. S4). When HeLa cells were transfected with a PERK expression vector, transcription from AARE (an enhancer element regulating the PERK pathway) was increased (lanes 2 and 3). In contrast, neither transcription from PGSE-A nor PGSE-B was upregulated but rather reduced by PERK overexpression. Thus, Fig. S4 and Fig. 5 indicate that all of three response pathways of the mammalian ER stress response are not involved in regulation of the PG pathway.

## Discussion

**Fig. 12:**
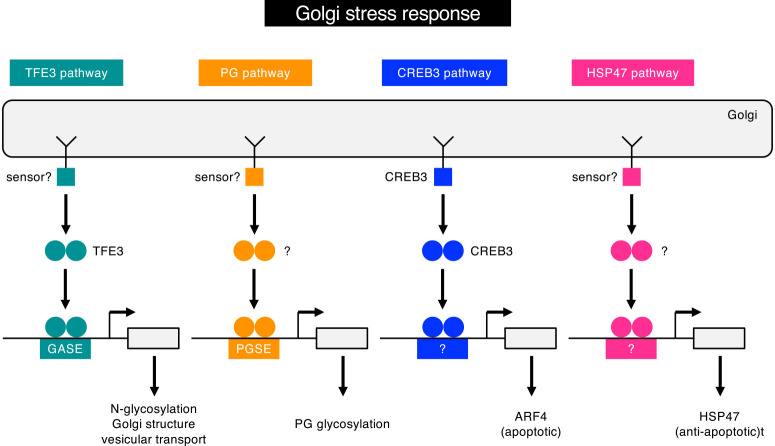
The current working hypothesis for the mammalian Golgi stress response. This study revealed a novel response pathway of the mammalian Golgi stress response, the PG pathway. The details are described in the text.

Here, we identified a novel response pathway in the mammalian Golgi stress response, the PG pathway (Fig. 12). This pathway is activated when PG glycosylation becomes insufficient (PG-Golgi stress), and upregulates transcription of genes involved in glycosylation of PGs in the Golgi. Promoter analyses revealed that the enhancer elements PGSE-A and PGSE-B regulate the proteoglycan pathway, for which the consensus sequences are CCGGGGCGGGGCG and TTTTACAATTGGTC, respectively.

The consensus sequences for PGSE-A and PGSE-B include binding sites for Sp1 and NF-Y, respectively. However, as these transcription factors are known to regulate basal expression of many mammalian genes, they cannot be responsible for transcriptional induction of PG-related genes in response to Golgi stress. Indeed, knockdown of Sp1 expression did not affect transcriptional induction by xyloside treatment even though basal transcription of PCYT1A, a Sp1 target gene, was reduced. The consensus sequence of ERSE, the enhancer regulating the ER stress response, is CCAAT(N9)CCACG (Yoshida *et al.*, 1998). NF-Y constitutively binds to the CCAAT portion of ERSE and only weakly increases transcription of target genes, whereas pATF6(N) binds to the CCACG portion in an ER stress-dependent manner and markedly induces transcription (Yoshida *et al.*, 2001). Therefore, it is possible that NF-Y constitutively occupies the CCAAT motif of PGSE-B and a stress-specific transcription factor binds the other portion of PGSE-B in order to upregulate transcription of GLCE.

Three mammalian Golgi stress response pathways have been reported: the TFE3, HSP47 and CREB3 pathways (Fig. 12). The target genes of the TFE3 pathway include the Golgi structural protein GCP60, N-glycosylation enzymes, such as SIAT4A, SIAT10, FUT1 and UAP1L1, and the vesicular transport components syntaxin 3A, RAB20, WIPI49, Giantin and GM130 (Oku *et al.*, 2011), which are involved in general Golgi functions. In contrast, target genes of the PG pathway include those encoding glycosylation enzymes for proteoglycans and do not overlap with those of the TFE3 pathway. We speculate that the TFE3 pathway ubiquitously regulates the general functions of the Golgi apparatus in different cell types, whereas the PG pathway specifically controls the capacity for PG glycosylation in the Golgi in PG-producing cells such as chondrocytes and reactive astrocytes. As the consensus sequences of PGSE-A and PGSE-B are different from that of GASE (ACGTGGC), and neither knockdown nor overexpression of TFE3 affected transcriptional induction by the PG pathway, the PG pathway is likely independent of the TFE3 pathway.

The HSP47 pathway is another mammalian Golgi stress response pathway identified by Tohyama and colleagues (Miyata *et al.*, 2013). The HSP47 pathway is activated by a chemical Golgi stress inducer, BenzylGalNAc, which leads to insufficient mucin-type glycosylation (mucin Golgi stress) by inhibiting glycosylation enzymes for mucins in the Golgi. Upon activation of the HSP47 pathway, expression of HSP47 is induced, which protects cells from Golgi stress-mediated apoptosis. HSP47 is an ER chaperone specializing in collagen folding (Nagata *et al.*, 1988), but it remains unclear how HSP47 inhibits apoptosis and how insufficient mucin glycosylation increases the expression of HSP47. The sensor, transcription factor and enhancer element regulating the HSP47 pathway have yet to be identified.

The CREB3 pathway, identified by Reiling and colleagues (Baumann *et al.*, 2018; Gendarme *et al.*, 2017; Ignashkova *et al.*, 2017; Ramirez-Peinado *et al.*, 2017; Reiling *et al.*, 2013; van Raam *et al.*, 2017), is activated by another Golgi stress inducer, brefeldin A, which inhibits vesicular transport from the ER to the Golgi. CREB3 is a sensor as well as key transcription factor regulating the CREB3 pathway, and belongs to the ATF6 family (Liang *et al.*, 2006). It is localized in the ER membrane as a transmembrane protein in the absence of Golgi stress, and upon Golgi stress or ER stress, it translocates from the ER to the Golgi. In the Golgi, the cytosolic domain of CREB3 is released from the membrane through proteolysis by site 1 and site 2 proteases, translocates into the nucleus, activates transcription of a small G protein, ARF4, and induces apoptosis. As expression of HSP47 and ARF4 mRNAs was reduced by 4MU-xyloside treatment according to the microarray, RNA seq and qRT-PCR analyses, the PG pathway is distinct from the HSP47 and CREB3 pathways.

The Golgi apparatus is more complicated in structure and function than the ER; therefore, the Golgi stress response likely has more pathways than the ER stress response, which has three pathways. Regarding the regulation of glycosylation, various glycosylation reactions occur in the Golgi. For example, N-glycans conjugated to proteins in the ER are further modified in the Golgi, and PG-type and mucin-type glycans are conjugated to proteins in the Golgi. In addition, lipids are also glycosylated in the Golgi. Thus, we speculate that the mammalian Golgi stress response comprises several response pathways, such as the mucin and the glycolipid pathways, which will be clarified in the near future. These response pathways are selectively activated depending on cellular demands and situations. For example, the PG pathway may be highly activated in chondrocytes producing many PGs, whereas the mucin pathway may be upregulated in mucin-producing cells such as goblet cells.

Recently, Shimizu and colleagues reported the GOMED (Golgi membrane-associated degradation) pathway of autophagy (Yamaguchi *et al.*, 2016). When vesicular transport from the trans-Golgi to plasma membrane is inhibited, the GOMED pathway is activated and degrades secretory proteins accumulated in the trans-Golgi via autophagy in an ATG5- and ATG7-independent manner. It is possible that GOMED is activated by an unknown Golgi stress response pathway, similar with ERAD being enhanced by the IRE1 pathway of the ER stress response.

Stimuli that activate these response pathways due to Golgi stress may be different among pathways. Insufficient capacity for PG glycosylation activates the PG pathway, whereas that for mucin-type glycosylation activates the mucin pathway. These different stimuli may be detected by distinct sensors for Golgi stress. Therefore, there are several types of Golgi stress, such as PG-type (insufficiency of PG glycosylation) and mucin-type (insufficiency of mucin-type of glycosylation), which separately activate a specific response pathway.

The method by which cells discriminate these different Golgi stress signals is important. During differentiation of PG-producing cells, synthesis of PG core proteins is increased and exceeds the capacity of glycosylation enzymes for proteoglycans, which may result in accumulation of incompletely glycosylated PG core proteins. Cells may interpret this accumulation as PG-Golgi stress. In the case of differentiation of mucin-producing cells, less glycosylated mucin core proteins may accumulate in the Golgi, which may be sensed by cells as mucin Golgi stress. However, it is difficult to discriminate less glycosylated PG core proteins from less glycosylated mucin core proteins. Goto and colleagues reported that different types of glycosylation occur in separate zones in the Golgi apparatus (Yano *et al.*, 2005). Therefore, cells may detect different types of Golgi stress (accumulation of different types of core proteins) by different sensors localized in different glycosylation zones in the Golgi.

Organelle autoregulation is one of the essential issues in cell biology, and research in this field, especially studies on ER stress response, mitochondrial unfolded protein response (Shpilka and Haynes, 2018), the Golgi stress response, the lysosomal stress response (Settembre *et al.*, 2013) and the peroxisome stress response (Yoshida, 2009), has recently increased. The core mechanism of these responses is similar. Sensors locating each organelle detect insufficiency of a specific organelle function and activate transcription factors, which increase expression of genes encoding proteins important for specific organelle functions via distinct enhancers. Identification of these regulators is critical for understanding organelle autoregulation.

In this study, we identified PGSE-A and PGSE-B, which are essential to identify the sensor and transcription factor regulating the PG pathway. The complete Golgi stress response mechanism will be revealed in the near future, which will advance our knowledge of cell biology as well as medical research for Golgi-related diseases.

## Supplementary Material

Fig. S1 Effect of xyloside treatment on morphology of organelles and cell shape. HeLa cells treated with 7.5 mM 4MU-xyloside or 6 mM 4NP-xyloside for 16 h were stained with DAPI and indicated antisera (A–C), or were observed by phase contrast microscopy (D). Bars=20 μm.

Fig. S2 Effect of xyloside treatment on CREB3 cleavage. Whole cell extract prepared from HeLa cells treated with 1 μg/ml brefeldin A (BFA), 1 μM MG132, 7.5 mM 4MU-xyloside or 6 mM 4NP-xyloside were subjected to immunoblotting with indicated antisera.

Fig. S3 PGSEs are active in cell lines other than HeLa cells. Murine embryonic fibroblasts (A) and C6 glioma cells (B) were transfected with indicated luciferase reporters fused with the indicated sequences, treated with 7.5 mM 4MU-xyloside for 18 h, and subjected to the luciferase assay. Values are means±SE of three independent experiments. ***, P<0.001; **, P<0.01; *, P<0.05.

Fig. S4 Transcription from PGSEs are not activated by PERK overexpression. HeLa cells were transfected with indicated reporter vectors and a PERK expression vector, and subjected to the luciferase assay. Values are means±SE of three independent experiments. ***, P<0.001; **, P<0.01; *, P<0.05.
