# Supplementary figures and images for: PGSE Is a Novel Enhancer Regulating the Proteoglycan Pathway of the Mammalian Golgi Stress Response
Source: Cell Struct Funct. 2018 Nov 28;44(1):1–19. doi: 10.1247/csf.18031 (PMC11926408; doi:10.1247/csf.18031)

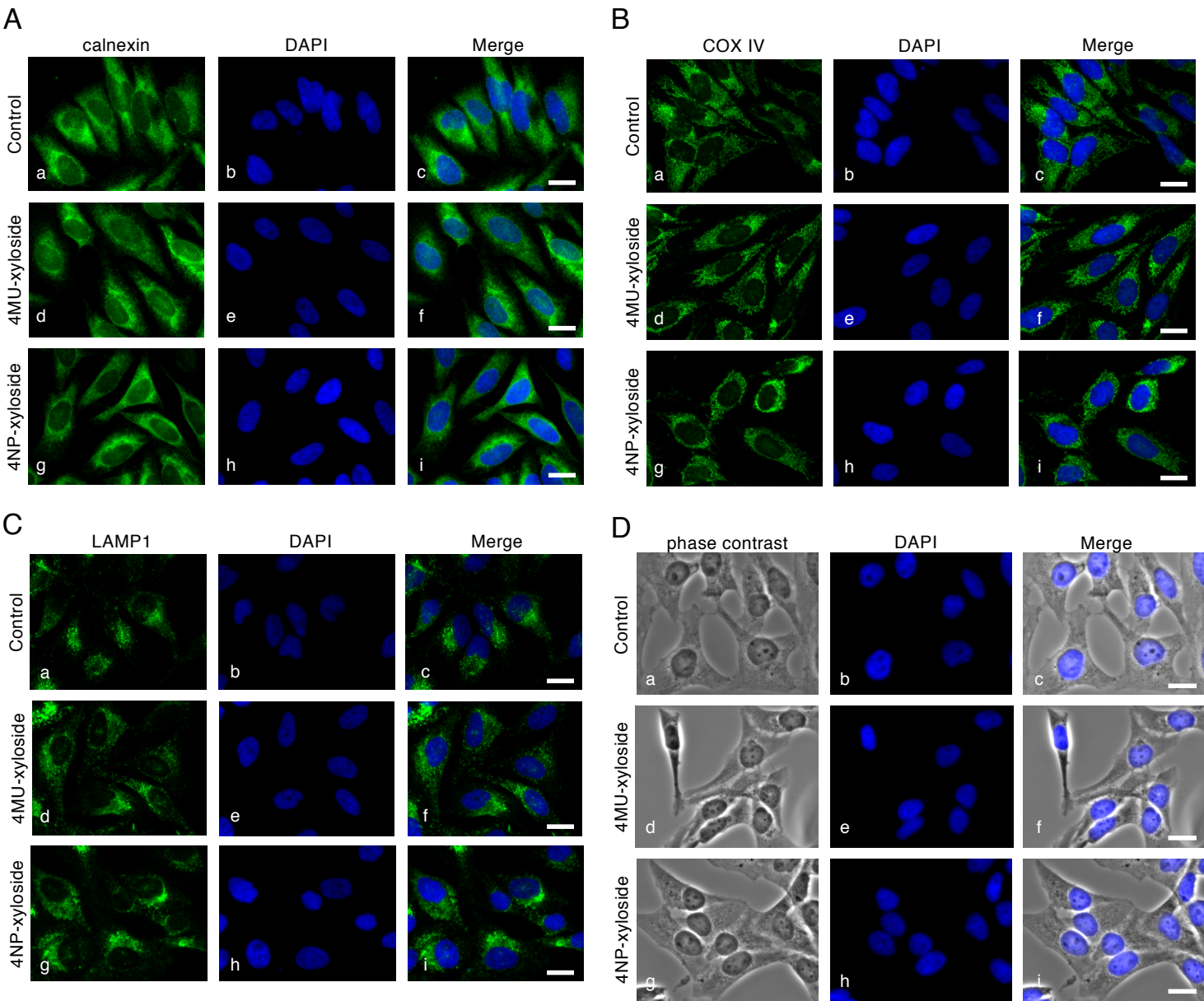

Supplement: Supplementary file 1 — Fig. S1 Effect of xyloside treatment on morphology of organelles and cell shape. HeLa cells treated with 7.5 mM 4MU-xyloside or 6 mM 4NP-xyloside for 16 h were stained with DAPI and indicated antisera (A–C), or were observed by phase contrast microscopy (D). Bars=20 μm. [file csf_44_18031_1.pdf]

**A**

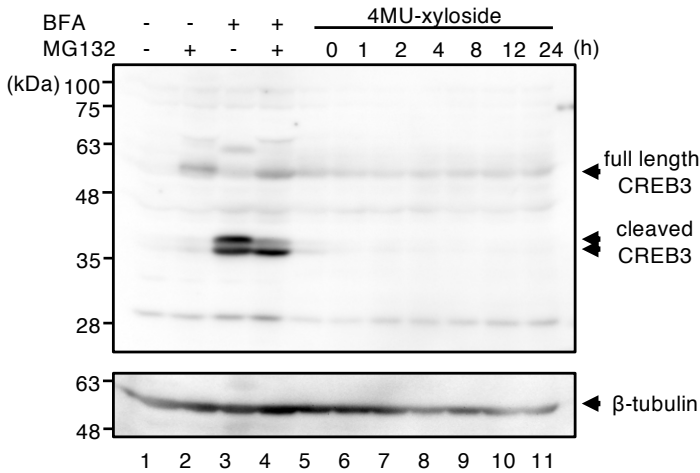

**B**

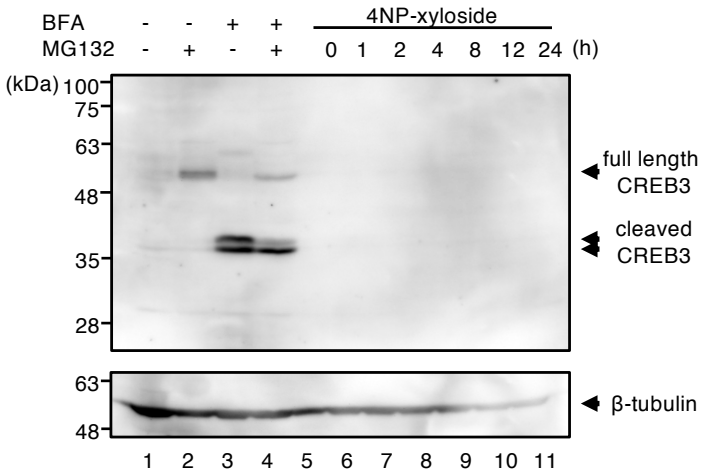

Supplement: Supplementary file 2 — Fig. S2 Effect of xyloside treatment on CREB3 cleavage. Whole cell extract prepared from HeLa cells treated with 1 μg/ml brefeldin A (BFA), 1 μM MG132, 7.5 mM 4MU-xyloside or 6 mM 4NP-xyloside were subjected to immunoblotting with indicated antisera. [file csf_44_18031_2.pdf]

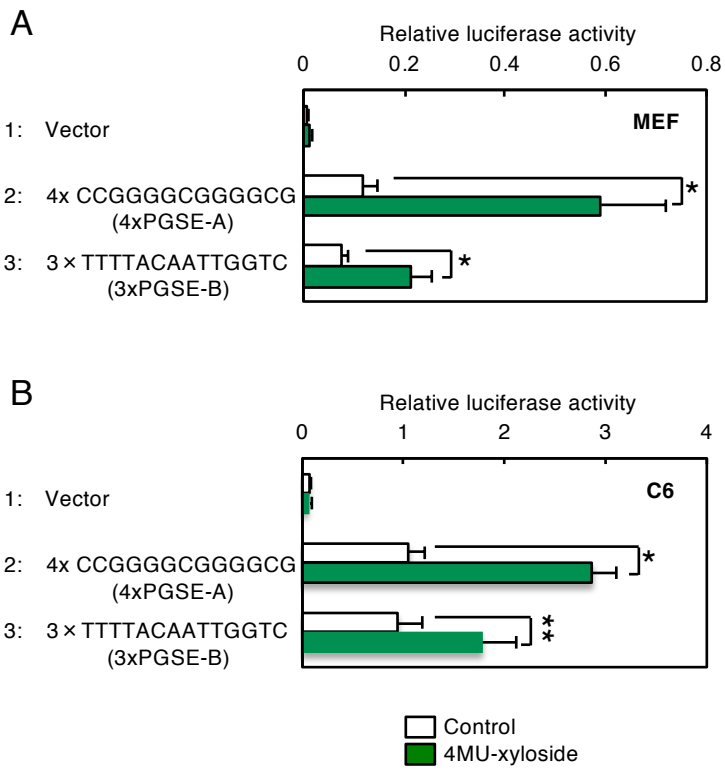

Supplement: Supplementary file 3 — Fig. S3 PGSEs are active in cell lines other than HeLa cells. Murine embryonic fibroblasts (A) and C6 glioma cells (B) were transfected with indicated luciferase reporters fused with the indicated sequences, treated with 7.5 mM 4MU-xyloside for 18 h, and subjected to the luciferase assay. Values are means±SE of three independent experiments. ***, P<0.001; **, P<0.01; *, P<0.05. [file csf_44_18031_3.pdf]

Figure S4 K. Sasaki et al.

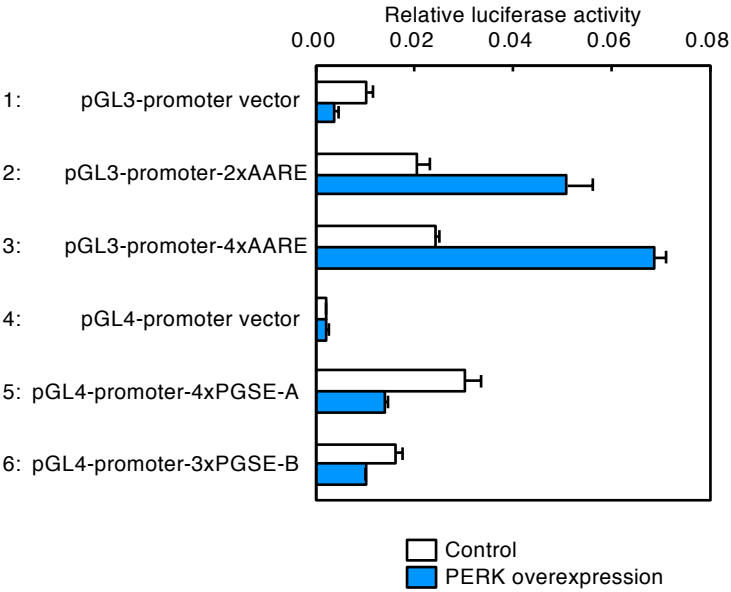

Supplement: Supplementary file 4 — Fig. S4 Transcription from PGSEs are not activated by PERK overexpression. HeLa cells were transfected with indicated reporter vectors and a PERK expression vector, and subjected to the luciferase assay. Values are means±SE of three independent experiments. ***, P<0.001; **, P<0.01; *, P<0.05. [file csf_44_18031_4.pdf]
